# Is There a Correlation between Apelin and Insulin Concentrations in Early Second Trimester Amniotic Fluid with Fetal Growth Disorders?

**DOI:** 10.3390/jcm12093166

**Published:** 2023-04-28

**Authors:** Dionysios Vrachnis, Nikolaos Antonakopoulos, Alexandros Fotiou, Vasilios Pergialiotis, Nikolaos Loukas, Georgios Valsamakis, Christos Iavazzo, Sofoklis Stavros, Georgios Maroudias, Periklis Panagopoulos, Nikolaos Vlahos, Melpomeni Peppa, Theodoros Stefos, George Mastorakos

**Affiliations:** 1 Department of Clinical Therapeutics, Alexandra Hospital, Medical School, National and Kapodistrian University of Athens, 115 28 Athens, Greece; 2 Third Department of Obstetrics and Gynecology, General University Hospital “Attikon”, Medical School, National and Kapodistrian University of Athens, 124 62 Athens, Greece; 3 First Department of Obstetrics and Gynecology, Alexandra Hospital, Medical School, National and Kapodistrian University of Athens, 115 28 Athens, Greece; 4 Department of Obstetrics and Gynecology, Tzaneio Hospital, 185 36 Piraeus, Greece; 5 Unit of Endocrinology, Diabetes Mellitus and Metabolism, Aretaieio Hospital, Medical School, National and Kapodistrian University of Athens, 115 28 Athens, Greece; 6 Department of Gynecologic Oncology, Metaxa Memorial Cancer Hospital, 185 37 Piraeus, Greece; 7 Second Department of Obstetrics and Gynecology, Aretaieio Hospital, Medical School, National and Kapodistrian University of Athens, 115 28 Athens, Greece; 8 Εndocrine Unit, 2nd Propaedeutic Department of Internal Medicine, Research Institute & Diabetes Center, General University Hospital “Attikon”, Medical School, National and Kapodistrian University of Athens, 124 62 Athens, Greece; 9 Department of Obstetrics and Gynecology, University of Ioannina, 45500 Ioannina, Greece

**Keywords:** apelin, insulin, amniotic fluid, second trimester, SGA, LGA, fetal growth, fetal macrosomia, FGR, fetal metabolism

## Abstract

Introduction: Fetal growth disturbances place fetuses at increased risk for perinatal morbidity and mortality. As yet, little is known about the basic pathogenetic mechanisms underlying deranged fetal growth. Apelin is an adipokine with several biological activities. Over the past decade, it has been investigated for its possible role in fetal growth restriction. Most studies have examined apelin concentrations in maternal serum and amniotic fluid in the third trimester or during neonatal life. In this study, apelin concentrations were examined for the first time in early second-trimester fetuses. Another major regulator of tissue growth and metabolism is insulin. Materials and Methods: This was a prospective observational cohort study. We measured apelin and insulin concentrations in the amniotic fluid of 80 pregnant women who underwent amniocentesis in the early second trimester. Amniotic fluid samples were stored in appropriate conditions until delivery. The study groups were then defined, i.e., gestations with different fetal growth patterns (SGA, AGA, and LGA). Measurements were made using ELISA kits. Results: Apelin and insulin levels were measured in all 80 samples. The analysis revealed statistically significant differences in apelin concentrations among groups (*p* = 0.007). Apelin concentrations in large for gestational age (LGA) fetuses were significantly lower compared to those in AGA and SGA fetuses. Insulin concentrations did not differ significantly among groups. Conclusions: A clear trend towards decreasing apelin concentrations as birthweight progressively increased was identified. Amniotic fluid apelin concentrations in the early second trimester may be useful as a predictive factor for determining the risk of a fetus being born LGA. Future studies are expected/needed to corroborate the present findings and should ideally focus on the potential interplay of apelin with other known intrauterine metabolic factors.

## 1. Introduction

Despite considerable scientific progress having been achieved in the field of fetal monitoring, fetal growth evaluation and surveillance are still challenging. Meanwhile, the underlying regulatory mechanisms continue to be under investigation given that it has long been known that fetal growth disturbances have a major impact on both short-term and long-term pregnancy outcomes. Perinatal morbidity and mortality are increased in small for gestational age (SGA) and large for gestational age (LGA) fetuses [1,2]. Growth-restricted fetuses (fetal growth restriction—FGR) that fail to reach their growth potential and the majority of severe cases of SGA fetuses (those below the 3rd centile) are most affected and at greater risk for adverse perinatal outcomes [3,4]. The same applies to fetuses with weight over the 90th centile partly due to labor complications [2,5,6]. Although the pathogenic mechanisms involved in both impaired and excessive fetal growth are still to be fully clarified, the majority of the existing literature on the topic implicates impaired uterine artery remodeling during early placental invasion and/or preterm placental insufficiency at a later gestational age [7,8].

In the current literature, several biomarkers have been investigated as possible markers of fetal growth aberrations, including apelin and insulin [9,10,11,12,13,14,15,16,17]. Apelin is an adipokine mainly produced in white adipose tissue and lung tissue, but also in the placenta. Apelin is encoded by the APLN gene located on the long arm of the X chromosome at position Xq25-26. Expression of the APLN gene produces pre-proapelin, which, after translational modification, is transformed into several apelin isoforms with different biological activities. A large number of published studies have highlighted the role of apelin in the cardiovascular as well as female reproductive systems. Interestingly, recent studies have investigated the expression of the apelinergic system in the placenta and its possible effects on specific pregnancy pathologies, such as preeclampsia, fetal growth restriction, and gestational diabetes mellitus [18,19,20,21,22,23,24,25].

Insulin is an essential hormone produced by the pancreas that contributes to the regulation of blood glucose levels. During pregnancy, maternal insulin production rises and exogenous administration may be needed to maintain normal maternal serum concentrations and prevent the consequences of gestational diabetes. Insulin is transferred to the amniotic fluid via fetal urine and its concentrations increase as pregnancy progresses. At present, there is some evidence demonstrating decreased amniotic fluid insulin concentrations in pregnancies complicated by placental insufficiency, fetal growth restriction, fetal malformations, or intrauterine fetal death [17]. Importantly, data exist showing that maternal glucose intolerance can impact the production of fetal insulin prior to 20 weeks gestation. Moreover, evidence published in the literature has pointed to an association between elevated amniotic fluid insulin concentration at 14–20 weeks gestation and both maternal glucose intolerance and fetal macrosomia, which were determined postnatally [26] Other studies have failed to reveal a correlation between amniotic fluid insulin concentrations and fetal growth [27].

This prospective observational study investigates the possible associations between apelin and insulin concentrations in the amniotic fluid of early second-trimester gestations with fetal growth abnormalities in the third trimester with regard to birthweight.

## 2. Subjects and Methods

### 2.1. Subjects

This is a prospective observational cohort study of 80 pregnant women consecutively recruited according to the inclusion criteria. The inclusion criteria were as follows: singleton pregnancies; pregnancies with indication for amniocentesis in the second trimester of pregnancy; advanced maternal age; increased nuchal translucency; and previous history of birth defects. The exclusion criteria were the following: pregnancies with major congenital abnormalities or chromosomal abnormalities as diagnosed by amniocentesis; multiple pregnancies; pregnancies occurring by in vitro fertilization; and pregnancies complicated by pregestational diabetes. All cases underwent amniocentesis after informed consent in the second trimester of pregnancy (15th to 22nd gestational week). Gestational age was estimated based on the date of the last period and was verified by a crown-rump length measurement taken from weeks 12 through 14. The maternal characteristics are shown in Table 1. Follow-up was carried out for all pregnancies until delivery. None of the participants classified within the AGA or the LGA groups were diagnosed with gestational diabetes during the current pregnancy, while only two cases belonging to the SGA group developed gestational diabetes out of the 80 women who were included in this study.

### 2.2. Protocol

At the first medical visit, the past medical histories of the pregnant women were taken. Following amniocentesis, amniotic fluid samples were collected. The latter were centrifuged immediately after amniocentesis and the supernatant was stored in polypropylene tubes at −80 °C. At delivery, neonatal birthweight was recorded, and gestational age-related fetal weight software allocated the exact weight centile of each fetus. Based on this calculation, the neonates were divided into three groups, as follows: fetuses with birthweight below the 10th centile (*n* = 31) were defined as small for gestational age (SGA); fetuses with birthweight between the 10th and 90th centile (*n* = 31) were defined as appropriate for gestational age (AGA); and fetuses with birthweight above the 90th centile (*n* = 18) were defined as large for gestational age (LGA). The group of AGA fetuses represents the control group. The study was approved by the Ethical Committee of Aretaieion University Hospital, Athens, Greece (143/291119), and was conducted in compliance with the Declaration of Helsinki guidelines.

### 2.3. Hormone Measurements

Amniotic fluid apelin concentrations were measured using the Apelin-12 (Human, Rat, Mouse, Bovine) extraction-free ELISA (enzyme immunoassay) kit (Phoenix Pharmaceuticals, Inc., Burlingame, CA, USA) according to the manufacturer’s instructions. Apelin is synthesized as the single peptide, preproapelin, which consists of 77 amino acids; these are converted into active fragments, including apelin-12, apelin-13, and apelin-36, which contain a range of amino acids formed by cleavage at specific sites. Most are bioactive. Standard immunoassays quantify apelin bioactivity as a whole and cannot specifically quantify each apelin peptide. The cross-reactivity of the kit for human peptides Apelin-12, Apelin-13, and Apelin-36 is 100%. The sensitivity concentration of the kit is 0.07 ng/mL, with a linear range 0.07–0.79 ng/mL, while the intra- and interassay variation is less than 10% and 15%, respectively. This ELISA kit has been used for human serum/plasma/cerebrospinal fluid or tissue extraction. Given the resemblance of early second-trimester composition to that of serum, it was appropriate for use in amniotic fluid. Amniotic fluid insulin concentrations were measured using the Quantikine^®™^ human insulin enzyme immunoassay (ELISA) kit (R&D Systems Inc., Minneapolis, MN, USA), according to the manufacturer’s instructions. The sensitivity concentration of the kit is 2.15 pmol/L, with a linear range 15.6–500 pmol/L, while the intra- and interassay variation is less than 4% and 8%, respectively. 

### 2.4. Statistics

Data were analyzed using the Statistical Package for Social Sciences (SPSS) version 21 (IBM Corp., Armonk, NY, USA; Released 2012. IBM SPSS Statistics for Windows, Version 21) [18]. Assessment of the normality distributions of the quantitive variables was carried out via graphical methods and Kolmogorov–Smirnoff analysis. The Mann–Whitney non-parametric test was employed for the comparison of continuous variables due to their abnormal distribution. For the categorical variables, the chi-square test was used with Fisher`s exact test as fewer than five observations were available. Differences were considered statistically significant if the null hypothesis could be rejected with >95% confidence (*p* < 0.05). Multiple regression analysis was used to define the independent effect of maternal age, weight, height, parity, gestational age at delivery, fetal sex, and amniotic fluid apelin and insulin concentrations on the possibility of a SGA/AGA or LGA/AGA birth. The Enter method was used for the analysis.

## 3. Results

### 3.1. Anthropometrics

No statistically significant difference was detected between the three studied groups (SGA, LGA, and AGA) with regard to maternal weight, height, BMI, or gestational age at birth. Maternal age, fetal sex, and fetal birthweight were significantly different among these groups (*p* < 0.05) (Table 1).

### 3.2. Apelin and Insulin Concentrations in Amniotic Fluid Samples in Relation to Fetal Growth

**Figure 1:**
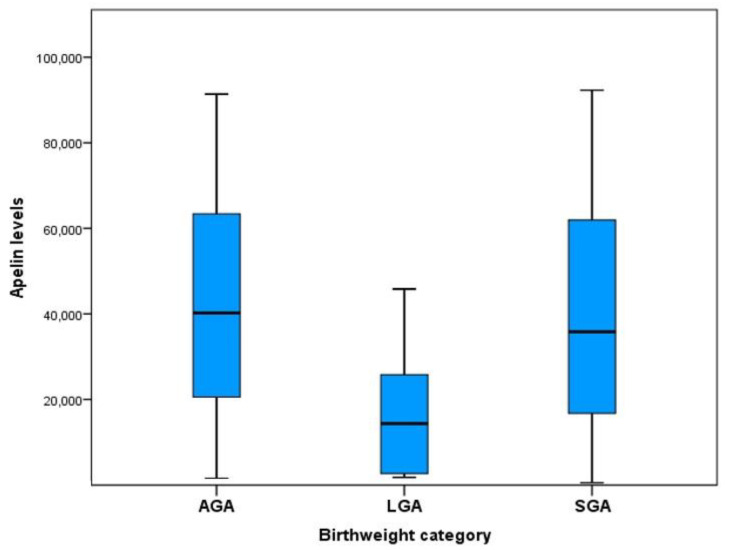
Apelin concentrations in the amniotic fluid of the AGA, SGA, and LGA groups. Box and whisker plot indicates box limits: Q1 and Q3.

Apelin and insulin concentrations in amniotic fluid were examined for potential differences among the three studied groups. The apelin concentrations for each of the three groups are presented in Table 2. Significantly lower concentrations of apelin were observed in LGA fetuses > 95th percentile compared to AGA fetuses. Differences among LGA fetuses > 97th percentile and AGA fetuses were not significant. This finding might be influenced by the small sample size of this group. No differences in apelin concentrations were observed between the SGA and AGA fetuses. Statistically significant differences were found among all the studied groups regarding apelin concentrations (*p* = 0.007). More specifically, apelin concentrations were significantly different between the AGA and LGA groups (*p* = 0.002), while there was no difference in apelin concentrations between the AGA and SGA groups (*p* = 0.668) (Figure 1). Insulin concentrations did not differ significantly among the three groups.

The possible associations between apelin concentrations in amniotic fluid and the severity of fetal growth disturbances were investigated. Table 2 presents the apelin concentrations in the amniotic fluid of pregnancies divided into subgroups according to SGA centile (3rd, 5th), AGA, and LGA centile (95th, 97th). Amniotic fluid apelin concentrations in the SGA fetuses were found to be greater than those in the AGA and LGA fetuses. Apelin concentrations progressively increased as the SGA centiles dropped. The apelin concentrations in the LGA fetuses were significantly lower compared to those in both the SGA and AGA fetuses (*p* = 0.015). By contrast, SGA fetuses below either the 3rd or the 5th percentiles exhibited greater apelin concentrations when compared with those in the AGA group (44.3, 41.5 and 40.2 ng/mL, respectively); however, the differences were not statistically significant.

In addition, the possible influence of fetal birthweight on the concentration of insulin in amniotic fluid was investigated. Table 3 illustrates insulin concentrations in amniotic fluid at the extremes of fetal birthweight. No statistically significant differences were detected between insulin concentrations in the amniotic fluid of the different fetal growth subgroups.

### 3.3. Predictors of SGA, AGA, and LGA Status among Maternal Anthropometrics, Fetal Sex, Gestational Age, and Amniotic Apelin and Insulin Concentrations

Multiple logistic regression of independent parameters such as maternal age, maternal weight, maternal height, fetal sex, and gestational age, which could influence the development of SGA, AGA, or LGA (dependent parameters), revealed that fetal female sex and amniotic insulin concentrations were significantly predictive of LGA fetuses (*p* = 0.047 and 0.042, respectively). The pseudo-R^2^_Nagelkerke_ of the regression analysis was 0.247 for the LGA vs. AGA analysis and 0.195 for the SGA vs. AGA analysis. Table 4 summarizes the results of the multiple regression analysis.

## 4. Discussion

Despite the considerable advances achieved in prenatal medicine, fetal growth abnormalities remain one of the most common causes of maternal and fetal mortality and morbidity; the underlying pathogenesis remains unclear and further investigation is certainly required. The present prospective observational cohort study was conducted in order to determine whether there is any correlation connecting the amniotic fluid apelin and insulin concentrations with fetal growth and birthweight abnormalities. This is, to the best of our knowledge, the first study to scrutinize apelin concentrations in the amniotic fluid of fetuses in the second trimester of pregnancy. The published data concerning apelin in pregnancy include studies in which the bioactive peptide was collected from either maternal blood or placental tissue [16,19,20,21,22,23,24,25,28]. Amniotic fluid in the early second trimester of pregnancy reflects fetal serum; therefore, amniotic fluid apelin concentrations correspond to fetal serum apelin concentrations [29,30].

In this study, we found that apelin concentrations were significantly lower in LGA fetuses compared to those in AGA and SGA fetuses, respectively. More specifically, the median apelin concentration in LGA fetuses was found to be 14.35 ng/mL, while in AGA it was 40.2 ng/mL, and in SGA it was 35.8 ng/mL. A progressive increase in apelin concentrations was observed with a reduction in birthweight, even though statistical analysis failed to show any significant difference. On the other hand, a significant effect was observed with increasing birthweight, which implies that the impact of apelin might not be evident until a critical fetal body mass has been attained. When regression analysis was conducted to account for confounding factors, the effect of apelin levels on fetal growth failed to remain significant. It is common that weak correlations, although biologically relevant, may be masked in regression analysis when several factors are included, especially in the case of small study samples. We believe that our study sample is responsible for this result. Moreover, none of the participants in the AGA or the LGA group were, later in pregnancy, diagnosed with gestational diabetes, a variable that can potentially affect fetal growth. Since a statistically significant association was identified between these two groups, gestational diabetes could not have been a factor influencing the results.

Several studies have indicated the positive effect of the apelinergic system on the proliferation of placental cells and trophoblast survival. These processes are essential during the second trimester when fetal growth is determined mainly by cell proliferation [4]. In their study, Van Mieghem et al. investigated apelin concentrations in maternal blood at several gestational ages: their findings revealed a 30% decrease in apelin concentrations in pregnancies with fetal growth restriction compared to normal pregnancies. Moreover, they highlighted that these serum results were also reflected in decreased placental apelin expression and staining. However, since their study included only four IUGR pregnancies their outcomes should be interpreted with caution [11]. In the present study, no such differences were revealed between AGA and SGA amniotic fluid apelin concentrations.

Apelin, which is an adipokine secreted by adipose and other tissues, shows elevated expression in obesity; it plays a central key role in lipid and glucose metabolism and is also implicated in atherosclerosis and oxidative stress. It is of note that pregnancy itself is characterized by hyperlipidemia, oxidative stress, and reduced insulin sensitivity [31,32]. Fetal macrosomia is considered to be the manifestation of an impaired maternal metabolism. Interestingly, cord blood apelin-36 levels are found to be similar in diabetic pregnancies compared to controls [33]. Apelin may be a mediator of fetal growth, but may also serve in protective feedback mechanisms; our finding of reduced apelin concentrations in LGA fetuses compared to AGA and SGA fetuses supports this hypothesis.

Regarding insulin concentrations, no statistically significant differences between the AGA, SGA, and LGA groups were found in the present study. However, paired multivariate regression analysis carried out for the AGA and LGA fetuses revealed that amniotic fluid insulin concentrations comprise an independent factor that affects fetal birthweight. In the past, we have shown that the low demand for nutrient uptake in the second trimester as well as the immaturity of the fetal pancreas could account for the lack of pronounced differences in insulin concentrations [11].

Reviewing the existing literature concerning insulin, the data are controversial. There is evidence that, prior to 20 weeks gestation, fetal insulin production may be impaired by maternal glucose intolerance. Interestingly, an association has been shown between increased amniotic fluid insulin concentration occurring at 14–20 weeks gestation and maternal glucose intolerance and fetal macrosomia observed postnatally [26]. On the other hand, other studies have failed to reveal a correlation between amniotic fluid insulin concentrations and fetal growth [27]. Other data show that prior to routine screening for gestational diabetes mellitus (GDM), exposure of the fetus to altered amniotic fluid glucose, insulin, and insulin-like growth factor-binding protein 1 has occurred [34]. Although a statistically significant difference was expected at least among severe LGA fetuses, subgroup analysis also failed to reach statistical significance, while a trend for higher values was observed. The small size of the specific subgroups may be responsible for this non-statistical significance.

Amniotic fluid insulin is known to be elevated in mothers with GDM versus those without [35,36,37]. It has also been hypothesized that insulin levels may be more closely associated with glucose intolerance rather than with growth disturbances, and glucose intolerance may be present without overt macrosomia; likewise, mild macrosomia may be present without significant insulin resistance. Hence, previous studies have already reported the considerable importance of amniotic fluid insulin levels as a predictor of fetal macrosomia in mothers suffering from gestational diabetes, as well as the fact that higher concentrations of amniotic fluid insulin levels are a marker of fetal hyperinsulinemia [38]. It is clear that identifying a hyperinsulinemic fetus before birth could lead to intensified maternal insulin therapy, thus reducing both the incidence and severity of diabetic fetopathy (a hormonal and metabolic dysfunction and its morphological sequelae) for the fetus of the diabetic mother.

A limitation of the present study is the small number of cases included, resulting in the small number of cases in the study subgroups. Of note, amniotic fluid is a biological material that is hard to collect, and thus, gathering sufficient cases prospectively is extremely difficult. For the same reason, we did not divide our cases further into subgroups according to the time of the amniocentesis. The exact gestational week of amniocentesis was defined by the indication. Moreover, our purpose was to correlate fetal development at term with apelin and insulin amniotic fluid levels in the early second trimester as a period of pregnancy and not by pregnancy week-by-week. This also allowed for the expansion of the implementation of our findings into clinical practice where the timing of invasive testing is mainly determined by the gestational stage at which the indication is set; this is in most cases after 16 weeks of gestation and usually up until 22–23 weeks, as per our study. Furthermore, we do not expect the levels of the studied substances to differ significantly by week during this time period, as this period of fetal life is characterized by a very shallow fetal growth curve. To the best of our knowledge, this is the first time that amniotic fluid apelin concentrations have been examined in the second trimester; this underlines the need for larger multicenter prospective studies to elucidate the possible associations between fetal growth abnormalities and amniotic fluid mediators, such as apelin and insulin, as well as their predictive value.

On the other hand, a strength of the study is the prospective design employed, which significantly limited the possibility of selection bias, consequently rendering our findings accurate and fully interpretable while being directly relevant to our population.

## 5. Conclusions

Amniotic fluid apelin concentrations in the early second trimester are likely to be useful as a predictive marker for the determination of the risk of a fetus being born LGA for gestational age. Whereas our study did not detect a statistically significant effect, a clear trend was identified toward decreasing values of apelin as birthweight progressively increased. It remains unknown whether other confounders may affect this association, including fetal gender, maternal age, and maternal weight, as the multiple regression analysis revealed that the coefficient of apelin for the detection of SGA and LGA compared to AGA was rendered non-significant. Larger studies are necessary to corroborate our findings and further expand on variables that could determine the variation of apelin levels; the studies should ideally focus on the potential interplay of apelin with known factors that appear to be predictive of LGA fetuses, including fasting maternal blood glucose concentrations, fasting insulin concentrations, and glucose concentrations in the oral glucose tolerance test. Such an approach could reveal the mechanisms that determine fetal growth and help us to better understand the pathophysiological pathways that place fetuses at risk of being born SGA or LGA.

## Figures and Tables

**Table 1:** Maternal characteristics and neonatal birthweight among the study groups. Maternal age, maternal weight, maternal height, maternal parity, fetal sex, gestational age in weeks, and birthweight are expressed using the median (25th quartile–75th quartile); statistical significance was set at *p* < 0.05 (bold values). Discrete variables were analyzed with the chi-square test using Fisher’s exact test; continuous variables were analyzed with the Mann–Whitney non-parametric test, as described in Section 2.

	AGA (*n* = 31)	LGA (*n* = 18)	SGA (*n* = 31)	*p*-Value
Maternal age (years)	35 (32–37)	35 (32–37)	37 (36–38)	**0.01**
Maternal weight (kg)	61.5 (56.25–72)	60.5 (55–64.75)	66 (59–78.5)	0.15
Maternal BMI	22.5 (18.4–34.1)	22.0 (18.5–29.6)	23.9 (17.9–40.3)	0.82
Maternal height (cm)	167 (165–171.5)	166 (158–170)	168 (163–170)	0.60
Maternal parity	1 (0–1.5)	1 (0–1)	0 (0–1)	0.24
Fetal sex (female)	11 (36.7%)	4 (23.5%)	19 (63.3%)	**0.02**
Gestational age (week)	38 (37–39)	38 (37–39)	38 (38–39)	0.11
Birthweight (gr)	3300 (3200–3510)	3870 (3667–4185)	2580 (2420–2775)	**0.01**

AGA: appropriate weight for gestational age, LGA: large weight for gestational age, SGA: small weight for gestational age, BMI: body-mass index.

**Table 2:** Comparisons of apelin concentrations (median, 25th quartile–75th quartile) of the study subgroups with apelin concentrations of AGA. The asterisk indicates a statistically significant difference from the AGA group (the asterisk indicates the statistical significance). Statistical significance was set at *p* < 0.05.

	No. of Cases	Apelin (ng/mL)
SGA < 10th centile	31	35.80 (15.60, 63.40)
SGA < 3rd centile	16	44.30 (11.28, 70.08)
SGA < 5th centile	22	41.50 (8.82, 62.88)
AGA	31	40.20 (18.90, 63.40)
LGA > 90th centile	18	14.35 * (2.59, 26.075)
LGA > 95th centile	11	17.90 * (2.71, 25.80)
LGA > 97th centile	4	21.15 (5.48, 33.30)

**Table 3:** Insulin concentrations (median, 25th quartile–75th quartile) did not differ among the study subgroups.

	No. of Cases	Median (Q1–Q3) (pmol/L)
SGA < 10th centile	24	2.265 (2.00, 3.59)
SGA < 3rd centile	12	2.34 (2.01, 4.45)
SGA < 5th centile	16	2.34 (2.00, 3,75)
AGA	27	2.40 (2.00, 2.88)
LGA > 90th centile	15	2.24 (2.00, 3.74)
LGA > 95th centile	9	2.68 (2.16, 3.78)
LGA > 97th centile	3	2.68 (2.13, 3.75)

**Table 4:** Multiple regression analysis of parameters with potential influence on fetal growth in SGA and AGA taken together, and LGA and AGA taken together. Statistical significance was set at *p* < 0.05 (bold value). AGA was used as the reference variable and the OR presented relates to the possibility of SGA or LGA for each examined variable.

	SGA and AGA		LGA and AGA	
Maternal age	1.17 (0.88, 1.56)	*p* = 0.276	0.81 (0.63, 1.05)	*p* = 0.109
Maternal weight	1.03 (0.94, 1.13)	*p* = 0.504	0.95 (0.89, 1.01)	*p* = 0.075
Maternal height	1.18 (0.97, 1.14)	*p* = 0.105	1.00 (0.86, 1.15)	*p* = 0.957
Maternal parity	0.83 (0.29, 2.40)	*p* = 0.725	0.93 (0.44, 1.96)	*p* = 0.853
Fetal female sex	8.37 (0.47, 150.27)	*p* = 0.149	0.21 (0.04, 0.98)	*p* = 0.047
Gestational age	1.87 (0.87, 4.05)	*p* = 0.110	1.26 (0.70, 2.26)	*p* = 0.449
Amniotic apelin	1.03 (0.98, 1.08)	*p* = 0.236	1.00 (0.97, 1.03)	*p* = 0.848
Amniotic insulin	1.38 (0.41, 4.57)	*p* = 0.601	0.61 (0.33, 0.94)	***p*** **= 0.042**

AGA: appropriate weight for gestational age, LGA: large weight for gestational age, SGA: small weight for gestational age.

## Data Availability

The data presented in this study are available on request from the corresponding author.
